# The Efficacy of a Newly Developed Cueing Device for Gait Mobility in Parkinson's Disease

**DOI:** 10.1155/2022/7360414

**Published:** 2022-05-18

**Authors:** Areerat Suputtitada, Carl P. C. Chen, Chatkaew Pongmala, Mana Sriyudthsak, Agnes Wilhelm, Pakpum Somboon, Jessie Janssen, Jim Richards

**Affiliations:** ^1^Department of Rehabilitation Medicine, Faculty of Medicine, Chulalongkorn University,and King Chulalongkorn Memorial Hospital, Bangkok, Thailand; ^2^Excellent Center for Gait and Motion, King Chulalongkorn Memorial Hospital, Bangkok, Thailand; ^3^Interdepartment of Biomedical Engineering, Graduate School, Chulalongkorn University, Bangkok, Thailand; ^4^Department of Physical Medicine and Rehabilitation, Chang Gung Memorial Hospital,Linkou and College of Medicine, Chang Gung University, Guishan, Taoyuan, Taiwan; ^5^Department of Neuroscience, University of Turin, Turin, Italy; ^6^Department of Electrical Engineering, Faculty of Engineering, Chulalongkorn University, Bangkok, Thailand; ^7^Department of Health Sciences, Institute of Therapeutic and Midwifery Sciences, IMC University of Applied Sciences Krems, Krems an Der Donau, Austria; ^8^Allied Health Research Unit, University of Central Lancashire, Preston, UK

## Abstract

**Background:**

External cues are effective in improving gait in people with Parkinson's disease (PD). However, the most effective cueing method has yet to be determined.

**Objective:**

The aim of this study was to compare the immediate effects of using visual, auditory, or somatosensory cues on their own or in combination during walking compared to no cues in people with PD.

**Methods:**

This was a single blinded, randomly selected, controlled study. Twenty people with PD with an age range of 46–79 years and Hoehn and Yahr scores of 1–3 were recruited. Participants were studied under 4 cueing conditions; no cue, visual, auditory, or somatosensory cues, which were randomly selected individually or in a combination.

**Results:**

A repeated measures ANOVA with pairwise comparisons using Bonferroni correction showed that any single or combination of the cues resulted in an improvement in gait velocity and stride length compared to no cue. Some significant differences were also seen when comparing different combinations of cues, specifically stride length showed significant improvements when additional cues were added to the light cue. The statistically significant difference was set at *p* < 0.05.

**Conclusions:**

Walking using visual, auditory, or somatosensory cues can immediately improve gait mobility in people with PD. Any or a combination of the cues tested could be chosen depending on the ability of the individual to use that cue.

## 1. Introduction

Abnormal gait patterns are commonly found in people with Parkinson's disease (PD). These often consist of short shuffling steps, increased cadence, decreased walking speed, and freezing of gait. Unfortunately, increased walking cadence, to compensate for the reduced step size, in combination with increased stride time variability is strongly correlated with risk of falling [1]. In addition, reduced stride length, one of the main characteristics of people with PD, is closely related to freezing of gait (FOG) [2, 3] and can lead to a loss of independence [4]. In addition, walking speed can be related to mobility, function, and mortality, which have all been shown to be decreased in people with PD [5] and disease progression, which in turn can lead to increase bradykinetic movements and a deterioration of gait, including increased frequency of FOG, and reduced postural control, which also increases the risk of falling [6].

Visual, auditory, and somatosensory cues have been shown to have some efficacies in improving gait mobility in people with PD [7–10], with visual cues significantly influencing stride length. Although, other authors have debated the effectiveness of visual cues, indicating that these had little or no effect on cadence or walking speed [8, 11]. Most reviews concluded that auditory cues are more effective in enhancing cadence and velocity; however, the effect on stride length is still debated. The effect of rhythmic somatosensory cues such as electrical stimulation and vibration [12–14] and insoles with a vibratory device [12, 15, 16] have also been explored. Rocha et al. concluded that somatosensory cues can increase walking speed, stride length, and decrease cadence [9]. Despite the evidence on different cue modalities, little is known about the effect of combining visual, auditory, and somatosensory cues, although some literature suggests that combined auditory and visual cues may improve cadence [10] and walking speed [9]; these findings are based on small sample sizes and have not explored all possible combinations of visual, auditory, or somatosensory cues.

Sweeney et al. [17] conducted a technical review of wearable cueing devices. Eighteen cueing systems were identified: five auditory cues, seven visual cues, three somatosensory cues, and a further three provided dual cueing modalities, two auditory and visual cueing systems, and one auditory and somatosensory cueing system. However, none of the devices combined all three cueing techniques. For this current study, a new device was developed which was able to produce all three external cues from one source. Therefore, the purpose of this study was to compare the immediate effects of using visual, auditory, or somatosensory cues on their own or in combination during walking compared to no cue in people with PD.

## 2. Materials and Methods

### 2.1. Research Design

**Figure 1:**
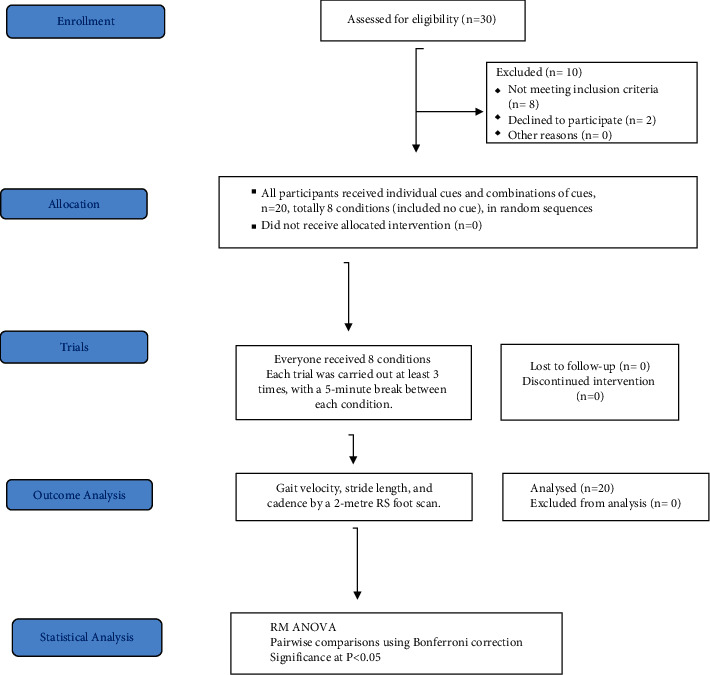
CONSORT flow diagram.

This study was randomly selected individually or in a combination, cross-over controlled trial with assessors and statisticians blinded. The flowchart for the Consolidated Standards of Reporting Trials (CONSORT) are shown in Figure 1.

### 2.2. Setting

Patients with Parkinson's disease were recruited at the outpatient rehabilitation clinic of King Chulalongkorn Memorial Hospital, Bangkok, Thailand, and the gait analysis was performed at the excellent center for gait and motion at the same hospital.

### 2.3. Participants

The inclusion criteria were patients with Parkinson's disease, aged between 45 and 80 years, with a Hoehn and Yahr stage between 1 and 3, able to walk for 10 meters without an assistive device and without assistance, currently taking antiparkinsonian medication, medically stable, with no vision impairment, no hearing impairment, no sensory impairment, and able to follow instructions. All participants were studied during the off period of Levodopa, the time which felt the worse symptom, and had positive of the pull test with score 1-2. All participants gave signed informed consent before taking part in the study, which was approved by the Institutional Review Board of the Faculty of Medicine, Chulalongkorn University, Thailand (IRB no. 51051).

### 2.4. The Newly Developed Cueing Device

**Figure 2:**
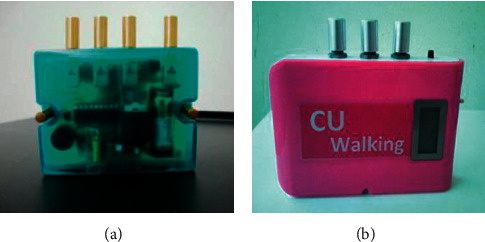
The newly developed cueing device (a) and the beautiful newly developed cueing device (b).

The cueing device used was developed by the authors. It consisted of 3 parts: visual, auditory, and somatosensory cueing, as shown in Figure 2(a), which was used in the research, and then, developed to improve the exterior design, as shown in Figure 2(b). The cueing device could be independently activated using an electronic switch by user selection, but it was done by the researcher in this study. In addition, the cueing rhythm could also be adjusted using a mobile application, and then, the setting value was sent from the mobile phone to the cueing device using Bluetooth communication. This cueing device is planned for using in telerehabilitation in the future. In this study, it was manual controlled done in-person by the researcher in the controlled environment at the excellent center for gait and motion. For visual cueing, a LED laser inside the device was used to generate a transverse line on the floor in front of the person. The position of this laser line could be adjusted according to the individual's step length, so that the line could clearly be seen while walking. An electronic buzzer was used to produce a sound for auditory cueing. Last, a vibration motor was used to provide somatosensory cueing. The in-system microcontroller and electronic circuits were used to control the sound and vibration levels of the auditory and the somatosensory cueing, respectively. Both auditory and somatosensory cues were set at 100 beats per minute.

### 2.5. Procedure

**Figure 3:**
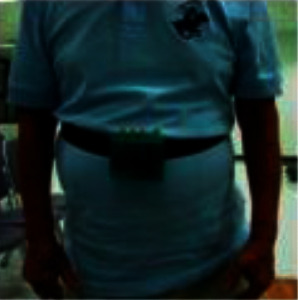
The device was placed on the abdomen and strapped around the waist.

The device was placed on the abdomen with strapped around the waist of the participant as shown in Figure 3. The participants were asked to walk at their own normal speed along a 10-metre long walkway. The researcher controlled each electronic switch without telling the participants. Each participant was asked to walk for eight trials, which were performed in a randomized order: no cue, visual cue, auditory cue, somatosensory cue, visual and auditory cues, visual and somatosensory cues, auditory and somatosensory cues, and visual, auditory, and somatosensory cues. The no cue trial was done by turning off the electronic switch despite the device was placed on the abdomen. The video demonstrated the procedure as in the supplementary.

### 2.6. Data Processing

**Figure 4:**
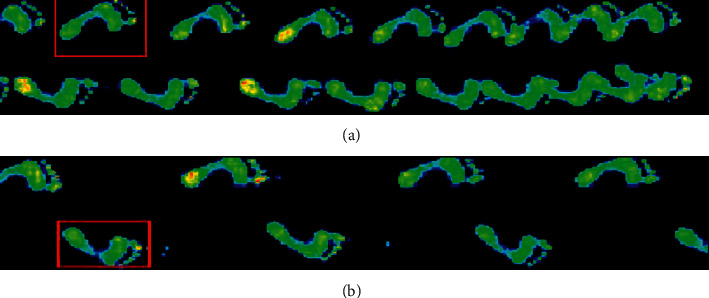
Example of the cadence measured during walking without (a) and with (b) using cues in a patient with Parkinson's disease. These figures were produced using 2-metre RS foot scan.

A 2-metre RS foot scan was embedded in the center of the walkway which was used to measure gait velocity, stride length, and cadence, as shown in Figure 4. Each trial was carried out at least 3 times, with a 5-minute break between each condition to minimize fatigue. The dependent variables for the analysis were velocity, stride length, and cadence.

### 2.7. Data Analysis

The data were found to be suitable for parametric testing using Shapiro–Wilk tests. Repeated measures analysis of variance (RM ANOVA) using a general linear model was used to examine the within-subject effects. For parameters with a significant main effect, between conditions analysis was conducted using pairwise comparisons using Bonferroni correction, and the effect sizes were calculated using partial eta-squared. A *p* value of less than 0.05 was used to determine statistical significance. All data were analyzed using SPSS version 22.

## 3. Results

Twenty patients with PD (11 women and 9 men) were recruited with a mean age of 66 ± 11.2 years (range 46–79), height of 1.57 ± 0.82 meters (range 1.45–1.70), weight of 56.1 ± 11.7 kg (range 40–74), BMI of 23.5 ± 5.2 kg/m^2^ (range 16.5–38.4), and a mean Hoehn and Yahr stage of 2.1 ± 0.9 (range 1–3). The RM ANOVA showed significant main effects for stride length, cadence, and velocity with effect sizes of (0.30–0.33), as given in Table 1. Further pairwise comparisons revealed significant differences in gait velocity, stride length, and cadence between no cueing device and all individual cues and combinations of cues. In addition, significant differences were seen when comparing different combinations of cues, specifically the step length was significantly greater when additional cues were added to the light cue, with the effects on cadence and walking speed showing less consistent findings (Table 2).

## 4. Discussion

This study showed that using any cue compared to no cue in patients with PD can lead to an improved walking speed and stride length and combining cues with the light cue can offer further improvements in stride length. Each cue may offer a different pathway in which it influences gait mobility [8]. This study is the first to combine all three cueing modalities in one device. Compared to no cue, combining light, sound, and/or a vibration cue can lead to a significant improvement in stride length and velocity. However, no additional benefits were seen when all three cues were combined compared to two cues. In addition, the cases recruited were in mild-to-moderate disease stages (H&Y 1–3); therefore, these were not cases with significant gait disorders (FOG, falls, and disabling postural instability). These findings are not widely applicable in all patients with PD.

Patients with PD do not lose the ability to move, but they tend to have a deficit in its activation. Several studies have supported the fact that using external cues is the most effective strategy to bypass these deficits [18, 19] due to external cues activating the premotor cortex, which is intact, rather than the basal ganglia/supplementary motor area circuit. However, there is a debate about the exact effect these cues have on gait parameters. The systematic review and meta-analysis by Magdi et al. [7] highlighted that cueing can be beneficial in improving functional activities and balance. The use of auditory, visual, and somatosensory cues leads to a statistically significant improvement in the step and stride length, speed of gait, and cadence, but it may not provide a significant change on gait parameters when compared to noncueing techniques [7]. The meta-analysis by Spaulding et al. [10] reported that auditory cueing can demonstrate significant improvements in cadence, stride length, and velocity. In contrast, visual cueing significantly improved stride length only [10]. A recent review verified the fact that cues can offer improvements in PD gait, improve psychomotor performance, and may also reduce freezing episodes. This study showed that visual and audio cues can significantly improve stride length and speed of gait; in addition, somatosensory cues also increase speed and stride length in patients with PD. We showed that all these cues, individually and combined, improved stride length and gait velocity. In contrast, this study showed no significant effect on cadence for any cues, or their combinations compared to no cue, except for the combination of sound and vibration.

Rocha et al. [9] made comparisons between visual, auditory, somatosensory, and a combination of visual and auditory cues. They concluded that visual cues can improve walking speed and decrease cadence, whilst auditory cues increase walking speed and step length, and somatosensory cues increase walking speed and stride length and decrease cadence. Although, in this study, we found that walking speed and stride length improved significantly in all cues used independently, the most effective single cue was the auditory cue for velocity. This may be explained by the auditory cue bypassing the internal rhythm deficit [8, 20], by providing a rhythm on a voluntary basis. When this rhythm is higher than normal walking speed, an increase in walking velocity might take place.

Only a few studies have considered the use of multiple cues simultaneously to improve walking parameters in people with PD [10]. The combination of visual and auditory cues led to improvements in cadence. Interestingly, this is not in agreement with this current study, where stride length proved to be most affected when a combination of visual and auditory cues was used. Visual cues have been shown to enable visual-cerebellar motor control and facilitate the generation of a better gait pattern [21], and the addition of sound could increase walking velocity. The systematic review by Spaulding et al. included different study designs: randomized clinical trials, nonrandomized, and cross-sectional, and only auditory and visual cues were considered [10]. They found that there were more than twice as many studies with auditory cues than with visual cues. This only included two randomized clinical trials with good methodological quality, and the other 23 studies were characterized as “preexperimental.” It was observed that the comparisons (type of cue versus type of training) produced significant improvements in gait speed compared with the use of interventions without cues or without any intervention.

Stride length appears to offer a clinically important outcome measure which was able to determine the differences between the cueing conditions. This agrees with McCandless et al. [19] found that the first and second step length changed with different cues during gait initiation. However, this current study also found that velocity and stride length also showed potentially clinically important changes between different combinations of cues. Therefore, future studies on gait initiation and freezing of gait could use these outcome measures to determine the effect of such interventions within clinical settings.

In this study, the frequency for sound and vibration was set at 100 beats per minute; however, it is currently unknown what the ideal frequency is to maximize the positive effect on gait mobility or whether individualized frequencies are more beneficial. A significant difference of 16% in stride length was seen when using light which had no control of cadence, and some of the changes in cadence when using sound and vibration could be explained by the 7% difference between the baseline cadence, which was not controlled, and the set beats per minute. In addition, the effect of one cue might have been carried over to the next cue, although a 5-minute rest was implemented between the different cues to counteract this.

## 5. Limitation and Further Suggested Studies

In this study, the acute or immediate effects of cueing were considered; however, Nieuwboer [22] reported that the immediate use of a cue may not produce the full effect of gait mobility, and longer periods of cued training may show more beneficial effects. Hence, future studies should ensure adequate training with cues and assistive devices and collect data regarding other presentations of PD, with the goal of characterizing subgroups and responders and nonresponders to different cueing conditions. In addition, for a patient with PD to be able to walk, other factors such as variability and stability of walking [23] are also important and need to be considered in further investigations. Moreover, this cueing should be further studied whether it could be used both at home and in the community, since the different setting may be some distraction as noise or spatiotemporal. When a device is going to be tested for use in patients with all further mentioned studies, it must be tested before and during with normal individuals as well.

## 6. Conclusion

This is the first study to combine all three cueing modalities in one device. Walking with individual visual, auditory, or somatosensory cueing devices or combination of two cues could immediately improve gait mobility in people with PD. Combinations of cueing methods are no more effective than using individual cues, except for additional cues to the light cue. Any or a combination of the cues could be chosen depending on the ability of an individual to use that cue or the given situation.

## Figures and Tables

**Table 1:** The outcome measures in 8 conditions.

Conditions	Patients with PD (*n* = 20)		
	Velocity (m/s)	Stride length (cm)	Cadence (strides/min)
No cue	0.61 (0.32)	69.12 (17.2)	51.53 (12.98)
Light	0.85 (0.34)	83.33 (21.6)	62.06 (11.79)
Sound	0.90 (0.28)	81.82 (20.0)	64.53 (9.21)
Vibration	0.82 (0.27)	84.61 (19.5)	59.71 (9.19)
Light and sound	0.88 (0.31)	80.86 (20.4)	61.23 (12.49)
Light and vibration	0.88 (0.29)	82.85 (19.7)	64.79 (12.78)
Sound and vibration	0.89 (0.29)	84.53 (21.5)	64.27 (12.19)
Light, sound, and vibration	0.87 (0.27)	84.5 (21.5)	61.89 (9.97)
ANOVA	*P* < 0.001^*∗*^	*P* < 0.001^*∗*^	*P*=0.001^*∗*^
Partial eta-squared *η*_p_^2^	0.33	0.33	0.182

^*∗*^Significant differences from the repeated measures analysis of variance (RM ANOVA) at *P* < 0.05.

**Table 2:** Pairwise comparisons of all conditions.

Comparisons	Velocity (m/s)		Stride length (cm)		Cadence (strides/min)	
	Mean difference	*P* value	Mean difference	*P* value	Mean difference	*P* value
No cue vs. light	−0.238	≤0.001^*∗*^	−11.1	≤0.001^*∗*^	−10.53	0.099
No cue vs. sound	−0.289	≤0.001^*∗*^	−14.2	≤0.001^*∗*^	−13.00	0.069
No cue vs. vibration	−0.211	≤0.001^*∗*^	−12.6	≤0.001^*∗*^	−8.18	1.000
No cue vs. light and sound	−0.266	≤0.001^*∗*^	−15.5	≤0.001^*∗*^	−9.70	0.562
No cue vs. light and vibration	−0.264	≤0.001^*∗*^	−11.7	0.023^*∗*^	−13.26	0.064
No cue vs. sound and vibration	−0.278	≤0.001^*∗*^	−13.7	≤0.001^*∗*^	−12.74	0.035^^*∗*^^
No cue vs. light, sound, and vibration	−0.261	≤0.001^*∗*^	−15.4	≤0.001^*∗*^	−10.36	0.210
Light vs. sound	−0.051	0.303	−3.0	0.019^*∗*^	−2.46	1.000
Light vs. vibration	0.027	0.605	−1.5	0.431	2.35	1.000
Light vs. light and sound	−0.028	0.624	−4.3	0.025^*∗*^	0.83	1.000
Light vs. light and vibration	−0.025	0.505	−0.5	0.876	−2.73	1.000
Light vs. sound and vibration	−0.040	0.377	−2.6	0.116	−2.20	1.000
Light vs. light, sound, and vibration	−0.023	0.607	−4.3	0.044^*∗*^	0.17	1.000
Sound vs. vibration	0.078	0.023^*∗*^	1.5	0.387	4.82	1.000
Sound vs. light and sound	0.023	0.608	−1.3	0.500	3.30	1.000
Sound vs. light and vibration	0.026	0.570	2.5	0.487	−0.26	1.000
Sound vs. sound and vibration	0.011	0.828	0.4	0.752	0.26	1.000
Sound vs. light, sound, and vibration	0.028	0.546	−1.3	0.549	2.64	1.000
Vibration vs. light and sound	−0.055	0.264	−2.8	0.093	−1.52	1.000
Vibration vs. light and vibration	−0.053	0.265	0.1	0.771	−5.08	1.000
Vibration vs. sound and vibration	−0.067	0.159	−1.1	0.371	−4.56	1.000
Vibration vs. light, sound and vibration	−0.050	0.227	−2.8	0.069	−2.18	1.000
Light and sound vs. light and vibration	0.003	0.944	3.8	0.163	−3.56	1.000
Light and sound vs. sound and vibration	−0.012	0.787	1.7	0.209	−3.04	1.000
Light and sound vs. light, sound, and vibration	0.005	0.901	0.0	0.979	−0.66	1.000
Light and vibration vs. sound and vibration	−0.015	0.621	−2.0	0.488	0.52	1.000
Light and vibration vs. light, sound, and vibration	0.003	0.934	−3.7	0.230	2.90	1.000
Sound and vibration vs. light, sound, and vibration	0.017	0.538	−1.7	0.144	2.38	1.000

^*∗*^Significant differences from the pairwise comparisons using Bonferroni correction at *P* < 0.05.

## Data Availability

The data presented in this study are available on request from the corresponding author.

## Supplementary Materials

Supplementary Materials Video 1. The participant walking with no cue. Video 2. The participant walking with light and sound cue.
